# Factors that may improve outcomes of early traumatic brain injury care: prospective multicenter study in Austria

**DOI:** 10.1186/s13049-015-0133-z

**Published:** 2015-07-16

**Authors:** Alexandra Brazinova, Marek Majdan, Johannes Leitgeb, Helmut Trimmel, Walter Mauritz, Austrian Working Group on Improvement of Early TBI Care

**Affiliations:** International Neurotrauma Research Organization (INRO), Mölker Gasse 4/3, 1080 Vienna, Austria; Department of Public Health, Faculty of Health Sciences and Social Work, Trnava University, Univerzitne nam.1, 91843 Trnava, Slovak Republic; Department of Traumatology, Medical University of Vienna, Spitalgasse 23, 1090 Vienna, Austria; Department of Anesthesiology, Intensive Care and Emergency Medicine, Wiener Neustadt Regional Hospital, Corvinusring 3-5, 2700 Wiener Neustadt, Austria; Department of Anesthesiology and Intensive Care Medicine, Trauma Hospital ‘Lorenz Boehler”, Donaueschingenstraße 13, 1200 Vienna, Austria

**Keywords:** Traumatic brain injury, Prehospital care, Early care, Emergency care, Comparative effectiveness research

## Abstract

**Background:**

Existing evidence concerning the management of traumatic brain injury (TBI) patients underlines the importance of appropriate treatment strategies in both prehospital and early in-hospital care. The objectives of this study were to analyze the current state of early TBI care in Austria with its physician-based emergency medical service. Subsequently, identified areas for improvement were transformed into treatment recommendations. The proposed changes were implemented in participating emergency medical services and hospitals and evaluated for their effect.

**Methods:**

14 Austrian centers treating TBI patients participated in the study. Between 2009 and 2012 all patients with Glasgow Coma Scale score < 13 and/or AIS head > 2 within 48 h after the accident, were enrolled. Data were collected in 2 phases: in the first phase data of 408 patients were analyzed. Based on this, a set of recommendations expected to improve outcomes was developed by the study group and implemented in participating centers. Recommendations included time factors (transport to appropriate trauma center, avoiding secondary transfer), adequate treatment strategies (prehospital fluid and airway management, anesthesia, ventilation), monitoring (pulse oximetry and blood pressure monitoring in all patients, capnography in ventilated patients) for prehospital treatment. In the emergency department focus was on first CT scan as soon as possible, short interval between CT scan and surgery and early use of thrombelastometry to optimize coagulation. Following implementation of these recommendations, data on 325 patients were collected and analyzed in phase 2. Final analysis investigated the impact of the recommendations on patient outcomes.

**Results:**

Patients in both data collection phases showed comparable demographic and injury severity characteristics. Treatment changes, especially in terms of fluid management, monitoring and normoventilation as well as thrombelastometry measurements were implemented successfully in phase 2, and led to significant improvement of patient outcomes. Hospital mortality was reduced from 31 % to 23 %. We found a lower rate of unfavorable outcomes, a significant increase in unexpected survivors and more patients with unexpected favorable outcomes as well.

**Conclusions:**

The results of this study clearly demonstrate that the outcomes of TBI patients can be improved with appropriate early care.

## Background

Traumatic brain injury (TBI) is still a leading cause of death and disability [1, 2], despite concentrated efforts during the last decades towards the improvement of prevention [3–6] and care. Globally, the incidence of TBI is increasing, mainly due to growing use of motor vehicles in low- and middle-income countries [7, 8] and to the aging of the population in developed countries [9]. As a common condition with serious consequences for the patient, it is important that treatment strategies are optimized, and it is known that the outcome of TBI patients might be improved by the implementation of, and adherence to, effective treatment guidelines [10, 11].

Evidence-based guidelines for treatment of TBI have been developed by the Brain Trauma Foundation (BTF) in 1995, and have first been published in 1996 [12]. A previous Austrian study (2002–2005) investigated the effects of compliance with these guidelines on outcomes of patients with severe TBI. This study revealed that nearly all of the standards, most of the guidelines, and some of the options mentioned in the BTF document had been followed in the participating Austrian hospitals [13]. Compliance with the recommendation for rapid resuscitation to achieve normal blood pressure and adequate oxygenation significantly improved Intensive Care Unit (ICU) survival (odds ratio (OR) 1.25 (CI 95 % [confidence interval] 1.12–1.39), and the rate of favorable outcome OR = 1.18 (CI95% 1.04–1.34). Based on this study we concluded that improved early TBI care will have an important role in achieving overall improvements in outcomes.

In Austria early TBI care in the field is done by emergency physicians whose primary specialty is in (almost 50 %) anesthesiology; internal medicine specialists, general practitioners and (trauma) surgeons participate in Emergency Medical Services (EMS), too. Prior to this project, some recommendations on prehospital treatment of TBI patients had been available in Austria; notably the guidelines on prehospital management of patients with TBI published by the BTF [14] in 2002 were incorporated into most of these local recommendations. However, these recommendations did not include treatment in the emergency room, which is mainly done by the anesthesiologists. The development and implementation of recommendations that included prehospital as well as early hospital care seemed thus warranted as it was usually physicians who treated patients in the field and in the emergency room, too.

The goals of this project were:

- ▪ To analyze the “status quo” of early TBI care in Austria, using “comparative effectiveness” as the main tool
- ▪ To define areas where a change of prehospital and early hospital treatment would improve outcomes
- ▪ To develop a set of recommendations for practical changes that were most likely to improve outcomes
- ▪ To implement the proposed changes in practice
- ▪ To analyze the effects of these changes

Our hypothesis was that we would be able to define areas for improvement, and that the proposed changes would improve outcomes of patients with moderate or severe TBI.

## Methods

The Austrian Ministry of Health and the Austrian Worker’s Compensation Board (AUVA) decided to fund the project, managed by the International Neurotrauma Research Organization (INRO). In 10/2008 all Austrian hospitals that treat patients with severe TBI were invited to participate in the study. 15 centers completed both data collection phases. One of them had very few patients in the second phase due to personnel issues. Therefore in final analyses we included 14 centers that participated in both phases and included patients according the inclusion criteria.

In Austria, out of approximately 120 hospitals treating any kind of traumatic brain injury (TBI) 30 hospitals treat more than 10 patients with severe TBI annually. According to patient load, hospitals participating in our study belong to this latter group.

In 11/2008, representatives from participating centers met in Vienna and founded the “Austrian Working Group on Improvement of Early TBI Care” (Working Group). The Working Group discussed and modified the study protocol prepared by INRO, and decided on a strategy for data collection. The Working Group made the following decisions used as inclusion criteria for the study:

- To enroll patients with moderate and severe TBI; these were defined as having a Glasgow Coma Scale score (GCS) <13 [15] without sedation within 48 h of the accident and/or having an Abbreviated Injury Scale (AIS) score of the region “head” >2 [16].
- To enroll all patients who were admitted during the 2 data collection phases. There were no age limits, and all patients fulfilling the defined criteria were to be enrolled, regardless whether they died in the trauma room or were transferred from another hospital.
- In-hospital patient management was supposed to be based on the 2007 version of the BTF guidelines on TBI treatment, and was to continue as usual in each center.
- To use of “off-line” documentation, in order to make this as simple as possible for the local study managers.

The study was approved by the ethical committees of all participating centers.

## First data collection phase (Phase 1)

Between 5/2009 and 4/2010 a total of 408 patients were enrolled by 14 participating centers. In each center one or more persons (local study manager) were in charge of patient enrollment. Information was collected on patient folders containing paper forms (mostly multiple-choice check-boxes, some fields for numbers, dates and times) for demographic and trauma data, prehospital status and treatment, trauma room status and treatment, computed tomography (CT) findings, data on surgical procedures, intensive care unit (ICU) status and treatment, and hospital outcome. Copies of relevant records (prehospital, trauma room, and anesthesia records, first laboratory results, DVDs with CT scan data) were included in these folders. Prehospital data were collected from Emergency Medical Services (EMS) protocols; any missing information was obtained from EMS by the local study managers and transcribed into study patients’ files. Each patient or a close relative gave written informed consent to agree to the follow-up.

The patient folders were collected at regular intervals by the project manager (AB) from the local study managers who were responsible for data collection. The data were then entered into an online database, created by INRO for this project. The database consisted of electronic transcript of patient folders used for data collection, separate sheets on Patient & Trauma data, Trauma Room data, CT & Operation data, Intensive Care Unit data, Outcome data. The online database was used for continuous data storage. In most cases data were entered on site, and missing data were obtained directly from the local study managers. In some cases data were entered in the INRO office, and missing data were obtained via phone call or e-mail. The DVDs with CT scan data were viewed by an intensive care specialist (WM), a radiologist, and a trauma surgeon (JL); judgement differences (e.g., with regard to the predominant injury) were resolved by a conference. All data collection forms were checked for completeness, and the local study managers were asked to provide missing information, if available. All information was checked for inconsistencies and/or implausible values; obvious errors were corrected. Six months after trauma the Extended Glasgow Outcome Scale (GOSE) score was obtained by phone interviews with patients and/or relatives and/or caregivers; this information was then entered into the database [17]. Ratings for the GOSE range from 1 to 8 (Dead to Upper Good Recovery). The 6-month outcome was dichotomized into unfavorable (GOSE score 1-6) and favorable (GOSE score 7–8) outcome groups.

### First data analysis

From the online database data were exported to an Excel™ file. Time intervals (EMS arrival – hospital arrival; hospital arrival – CT scan, CT scan- Operating Room, etc.), injury severity scores (ISS), and probabilities of mortality and of unfavorable 6-month-outcome were calculated. Outcome probabilities were calculated using the IMPACT core model [18] which has been validated for our sample [19]. Expected mortality and unfavorable outcome were calculated for the whole study group with all known values. Based on the calculated probabilities and real outcomes, ratios of observed versus expected mortality and of observed versus expected rates of unfavorable outcome (O/E ratios) were calculated (in the patient group with known outcome values). These O/E ratios were used to analyze the effectiveness of treatment options. Individual treatment options (e.g., use of monitoring, use of fluids, prehospital intubation, interval between hospital admission and CT scan, etc.) were analyzed by comparing O/E ratios of patients with or without a specific treatment option, respectively (“comparative effectiveness analysis”). A treatment option was considered to be effective if the O/E ratios of patients that had received this treatment were <1 (i.e., if rates of mortality and of unfavorable outcome were lower than expected). A treatment option was thus considered to be not effective if the O/E ratios of patients were >1 (i.e., if rates of mortality and of unfavorable outcome were higher than expected).

The differences between the numbers of observed and expected survivors (or observed and expected favorable outcome at 6 months after injury) were defined as “unexpected survivors“(or “unexpected favorable outcomes“).

### Development and implementation of recommendations

In 11/2010, the full Working Group met again, studied the results of the first phase data analysis, and developed recommendations for the early management of TBI patients. The important aspects of these recommendations included:

- time factors: fast transport to an appropriate center if field GCS score <13 or other signs of neurological impairment, first CT scan within 20–30 min after arrival, in cases of intracranial hematoma interval between CT scan and surgery <60 min.
- adequate monitoring: use of capnography in all ventilated patients, use of pulse oximetry, and use of blood pressure monitoring
- fluid resuscitation: avoid hypotonic solutions, use of Ringer’s solution or other balanced electrolytes solutions, consider hypertonic saline in hypotonic patients
- adequate ventilation: normoventilation (monitored by capnography)
- early thrombelastometry (TEM) to optimize coagulation in all patients >60 years and/or on anticoagulants
- medication: avoidance of steroids

This document (in German) was available via the INRO website (www.igeh.org), and was distributed to all centers. The local study managers of each center were responsible for convening meetings with the EMS providers in their regions to make the EMS staff familiar with the important aspects of these recommendations. All centers were supposed to implement these recommendations by 02/2011. In addition, the findings of phase 1 were presented at all national EMS, trauma- or anesthesia congresses in Austria in 2011.

### Second data collection phase (Phase 2)

The second data collection phase started in 04/2011 and lasted 10 months. This phase was shorter than the first one due to funding restrictions, however, the monthly patient enrollment rate was similar. Enrollment criteria and data collection were identical to the first phase. Data on 325 patients collected in 14 participating centers were analyzed. Again, patient folders were collected at regular intervals, data was entered as before, and GOSE scores at 6 months after the injury were obtained by phone interviews.

### Final data analysis

Data was exported from the online database to an Excel^TM^ file. As before, data were checked for completeness and consistency, implausible values were corrected, missing information was retrieved, and calculations (intervals, probabilities) were made. Differences in epidemiology, treatment and outcomes between Phase 1 and Phase 2 patients were analyzed.

### Statistical analysis

Means with respective standard deviations or medians with inter-quartile ranges were used as central measures of continuous variables, and counts with percentages were used as frequency measures of categorical variables. For the statistical analysis of continuous variables the Student’s *T*-test was used when analyzing means, and the Wilcoxon test when analyzing medians. For the analysis of categorical variables the Chi^2^-Test was used. In case of ‘Unknown’ (missing) cases the tests were applied only on known values. Logistic regression was used for multivariable analysis of factors influencing the outcome of patients. Variables that had significant influence on the outcome in univariate analysis were used for the logistic regression model. Treatment center was also used as a predictor, thus the presented results took the ‘center effect’ into the account. Odds ratios are presented with 95 % confidence intervals.

A *p*-value of <0.05 was considered statistically significant.

## Results

Demographic data of patients from Phase 1 versus Phase 2 are given in Table 1. The two groups were similar; there were no significant differences regarding mean age, male: female ratio, and mechanisms of trauma.

**Table 1:** Characteristics of study group: sex, age, mechanism of TBI, accident site, type of trauma

	Phase 1		Phase 2		Total		*p*-value*
	n	%	n	%	n	%	
Total	408		325		733		
Male	297	73	233	72	530	72	0.804
	**Mean**	**SD**	**Mean**	**SD**	**Mean**	**SD**	
Age	48	24	50	22	49	23	0.221
Injury Mechanism	**n**	**%**	**n**	**%**	**n**	**%**	
Traffic^a^	144	35	133	41	277	38	0.086
Fall < 3 m	123	30	92	28	215	29	
Fall >3 m	45	11	44	14	89	12	
Sports activity	43	11	16	5	59	8	
Work related	16	4	13	4	29	4	
Firearm	5	1	3	1	8	1	
Assault	2	0	4	1	6	1	
Other/Unknown	30	7	20	6	50	7	
Total	408	100	325	100	733	100	
Type of trauma	**n**	**%**	**n**	**%**	**n**	**%**	
Blunt	355	87	294	90	649	89	0.435
Penetrating	29	7	18	6	47	6	
Unknown	24	6	13	4	37	5	
Total	408	100	325	100	733	100	
Accident Site	**n**	**%**	**n**	**%**	**n**	**%**	
Home	108	26	79	24	187	26	0.032
Outdoor/sports	78	19	47	14	125	17	
Road/public area	168	41	153	47	321	44	
Workplace	32	8	12	4	44	6	
Unknown	22	5	34	10	56	8	
Total	408	100	325	100	733	100	

Legend: *SD* Standard Deviation

*The tests were applied only on known values

^a^Traffic contains: bicycle rider, motor vehicle driver/occupant, pedestrian injurred in traffic accident

Severity of trauma, CT scan findings, and expected outcomes are given in Table 2. The initial GCS was significantly higher in Phase 2 patients; the median, however, indicated that in both groups most patients had severe TBI. The ISS was also significantly higher in Phase 2 patients. The proportion of patients with prehospital hypotension and/or hypoxia was similar in both groups. Phase 2 sample had a significantly lower rate of patients with normal pupillary reflex. Regarding CT scan findings compressed or closed basal cisterns as well as greater midline shift were seen significantly more frequently in Phase 2 patients. The predominant type of brain lesion was not significantly different between the 2 groups. Phase 2 patients had higher trauma severity, worse CT findings, and worse pupillary reactivity. Expected mortality rates and the expected rates of unfavorable outcome were similar in both groups.

**Table 2:** Characteristics of study group: trauma severity, CT scan findings, expected outcomes

	Phase 1		Phase 2		Total		*p*-value*
	median	IQR	median	IQR	median	IQR	
GCS score	6	(3–6)	7	(3–11)	6	(3–11)	<0.05
ISS	25	(16–25)	29	(19–43)	26	(17–41)	<0.01
	**n**	**%**	**n**	**%**	**n**	**%**	
Field hypotension^a^	32	10	30	11	62	10	0.887
Field hypoxia	63	21	56	21	119	21	0.988
Pupils	**n**	**%**	**n**	**%**	**n**	**%**	<0.001
Both reactive	334	82	206	63	540	74	
One reactive	10	2	31	10	41	6	
None reactive	53	13	49	15	102	14	
Not assessable/Unknown	11	3	39	12	50	7	
Total	408	100	325	100	733	100	
CT scan: basal cisterns	**n**	**%**	**n**	**%**	**n**	**%**	<0.001
Open	315	77	204	63	519	71	
Compressed	57	14	56	17	113	15	
closed	27	7	41	13	68	9	
Unknown	9	2	24	7	33	5	
Total	408	100	325	100	733	100	
CT scan: midline shift	**n**	**%**	**n**	**%**	**n**	**%**	<0.001
No shift	286	70	203	62	489	67	
<5 mm	45	11	25	8	70	10	
>5 mm	40	10	75	23	115	16	
Unknown	37	9	22	7	59	8	
Total	408	100	325	100	733	100	
CT scan: brain lesions	**n**	**%**	**n**	**%**	**n**	**%**	
Subarachnoid hemorrhage	226	55	190	58	416	57	0.825
Subdural hematoma	213	52	166	51	379	52	0.869
Contusions	140	34	135	42	275	38	0.228
Intracerebral hematoma	78	19	68	21	146	20	0.701
Epidural hematoma	60	15	62	19	122	17	0.231
Intraventricular hemorrhage	35	9	33	10	68	9	0.6
Hemorrhage in basal cisterns	28	7	24	7	52	7	0.928
Predominant injury	**n**	**%**	**n**	**%**	**n**	**%**	
Normal CT	36	9	21	6	57	8	0.572
Contusions	38	9	39	12	77	11	
Diffuse edema	47	12	47	14	94	13	
Subarachnoid hemorrhage	68	17	43	13	111	15	
Epidural hematoma	37	9	29	9	66	9	
Subdural hematoma	129	32	105	32	234	32	
Intracerebral hematoma	39	10	26	8	65	9	
Intraventricular hemorrhage	8	2	7	2	15	2	
Unknown	6	1	8	2	14	2	
Total	408	100	325	100	733	100	
**Expected outcomes**	**mean (%)**	**SD**	**mean (%)**	**SD**	**mean (%)**	**SD**	
Probability of death^b^	33	20	34	21	33	20	0.8517
Probability of unfavorable outcome^a^	55	22	55	24	55	23	0.8517

Legend: *GCS* Glasgow Coma Scale, *ISS* Injury Severity Score [33], *IQR* Interquartile range, *CT* Computed Tomography

*The tests were applied only on known values

^a^Prehospital (field) hypoxia was defined as oxygen saturation <90 % (measured by a pulse oximeter) until hospital admission. Prehospital (field) hypotension was defined as systolic blood pressure <90 mmHg until hospital admission

^b^Probability of death/unfavorable outcome was calculated using IMPACT Core model [18] on the whole study group with known data (age, GCS motor score, pupils)

The treatment options that had been recommended based on association with improved outcomes are listed in Table 3. All of them were used more frequently during Phase 2. Direct transportation to the participating centers occurred significantly more frequently (87 vs. 78 %). Regarding time intervals, in Phase 2 significantly more patients reached the hospital within 60 min after EMS arrival (61 vs. 41 %), had their first CT scan within 60 min after hospital arrival (74 vs. 66 %), and – if required – had neurosurgical operations started within 120 min of hospital arrival (36 vs. 32 %). Prehospital monitoring (pulse oximetry, blood pressure monitoring, and capnography) was used more frequently in Phase 2. Prehospital ventilation was improved, as fewer patients were hyperventilated and more patients had optimal ventilation. Regarding prehospital fluid resuscitation Ringer’s lactate solutions as well as colloids (mainly hydroxy ethyl starch) were used less frequently, while Ringer’s and other balanced electrolyte solutions were used more frequently. Prehospital infusion volumes were not significantly different: 700 (SD 698 ml) in Phase 1 vs. 800 (SD 737 ml) in Phase 2, and were given on average within the time frame of EMS care (50 ± 25) minutes. Within in-hospital treatment in Phase 2 thrombelastometry (TEM) was used more frequently (in 40 % vs. 24 %), and corticosteroids were given significantly less frequently: 2 of the 3 cases that received corticosteroids during Phase 2 had concomitant spinal cord injuries, and the corticosteroids were used due to that.

**Table 3:** Recommended treatment options

	Phase 1		Phase 2		Total		*p*-value*
Transport	**n**	**%**	**n**	**%**	**n**	**%**	
Direct	317	78	282	87	599	82	<0.01
Secondary	70	17	32	10	102	14	
Unknown	21	5	11	3	32	4	
Total	408	100	325	100	733	100	
Time intervals	**n**	**%**	**n**	**%**	**n**	**%**	
EMS arrival - hospital admission <60 min	167	41	197	61	364	50	<0.01
Hospital admission - CT scan <60 min	269	66	242	74	511	70	0.854
Hospital admission - OR <120	131	32	118	36	249	34	0.995
Prehospital monitoring	**n**	**%**	**n**	**%**	**n**	**%**	
Pulse oximetry used	298	73	284	87	582	79	0.969
Blood pressure monitoring used	291	71	267	82	558	76	0.966
Capnography in ventilated patients	123	57	131	78	254	35	0.121
Prehospital ventilation	**n**	**%**	**n**	**%**	**n**	**%**	
Hyperventilation (pCO2 < 35 mmHg)	81	20	47	14	128	17	0.083
Normoventialtion (pCO2 35–42 mmHg)	64	16	59	18	123	17	
Hypoventilation (pCO2 > 42 mmHg)	10	2	13	4	23	3	
Unknown	253	62	206	63	459	63	
Total	408	100	325	100	733	100	
Prehospital fluid resuscitation	**n**	**%**	**n**	**%**	**n**	**%**	
Ringer's lactate	109	27	65	20	174	24	0.096
Ringer's solution	102	25	110	34	212	29	0.148
Other electrolyte solution	71	17	74	23	145	20	0.283
Colloids	105	26	59	18	164	22	<0.05
Hypertonic saline	22	5	15	5	37	5	0.665
Other treatment options	**n**	**%**	**n**	**%**	**n**	**%**	
Trombelastometry done	99	24	129	40	228	31	0.212
Corticosteroids given	15	4	3	1	18	2	0.045

Legend: *CT* Computed Tomography, *OR* Operation Room, *EMS* Emergency Medical Service, *pCO2* Partial Pressure of Carbon Dioxide

*The tests were applied only on known values

During Phase 2 neurosurgical procedures were required more frequently (67 % vs. 59 %), ICP was monitored more often (in 60 % vs. 51 %), but for shorter periods (8 [IQR 5–14] vs. 9 days [IQR 5–13]), and the duration of ventilation was longer (10 days [IQR 3–18] vs. 8 [IQR 2–15]). No significant differences between Phase 2 and Phase 1 were observed regarding rates of prehospital intubation (62 % vs. 60 %), of prehospital management by helicopter crews (48 % vs. 46 %), and of therapeutic hypothermia (14 % vs. 21 %).

Outcomes were significantly better in Phase 2 (Table 4). Hospital mortality was 22 % in Phase 2 vs. 29 % in Phase 1, and the rate of patients with unfavorable outcome was 32 % in Phase 2 vs. 38 % in Phase 1.

**Table 4:** Outcomes at hospital discharge and 6 months after trauma

	Phase 1		Phase 2		Total		*p*-value*
Survival	**n**	**%**	**n**	**%**	**n**	**%**	
TR deaths	9	2	4	1	13	2	0.1242
ICU deaths	106	26	67	21	173	24	<0.001
Hospital deaths	118	29	72	22	190	26	<0.001
6 m deaths	127	31	72	22	199	27	<0.001
Unknown at 6 m	179	44	39	12	218	30	
Total	408	100	325	100	733	100	
6 month outcome	**n**	**%**	**n**	**%**	**n**	**%**	
Favorable	75	18	181	56	256	35	<0.001
Unfavorable	154	38	105	32	259	35	
Unknown	179	44	39	12	218	30	
Grand Total	408	100	325	100	733	100	

Legend: *the tests were applied only on known values; 6 month outcome was measured by GOSE (Glasgow Outcome Scale extended) and dichotomized into favorable (GOSE score 7–8) and unfavorable (GOSE score 1–6) outcomes

Comparison of expected and observed outcomes is presented in Table 5. There were significantly more unexpected survivors as well as patients with unexpected favorable outcomes.

**Table 5:** Comparison of expected and observed outcomes at hospital discharge and 6 months after trauma

	Phase 1	Phase 2
Hospital (Outcome known) (n)	383	309
Expected Mortality (mean%)	34	34
Expected Deaths (n)	130	105
Observed deaths (n)	118	72
Observed mortality (mean%)	31	23
OE ratio	0,91	0,69
Unexpected survivors (n)	12	33
6 month total (Outcome known) (n)	229	286
Expected Unfavorable (mean%)	60,3	54
Expected Unfavorable (n)	138	154
Observed Unfavorable (n)	154	105
Observed Unfavorable (mean%)	67	37
OE ratio	1,12	0,68
Unexpected favorable (n)	-16	49

Legend: *OE* observed/expected; 6 month outcome was measured by GOSE (Glasgow Outcome Scale extended) and dichotomized into favorable (GOSE score 7–8) and unfavorable (GOSE score 1–6) outcomes, *OE* Observed versus Expected

Table 6 presents the results of multivariable analysis of factors influencing the outcome of patients. For the logistic regression model the variables that had significant influence on the outcome in univariate analysis were used. Patients in Phase 2 had significantly higher odds of ICU survival (OR 2.92, CI95%: 1.35–6.31) and hospital survival (OR 3.01, 1.41–6.38) and higher odds of favorable outcome (OR 3.14, 1.72–5.73). These findings have been adjusted for age, indicators of severity and treatment, and for “center effect” (by including the treatment center as one of the independent variables in the model).

**Table 6:** Multivariable adjusted analysis of factors influencing the outcome of patients^a^

Predictor	ICU Outcome (Alive = 1)		Hospital Outcome (Alive = 1)		Long Term Outcome (favorable = 1)	
	OR (CI 95 %)	*p*-value	OR (CI 95 %)	*p*-value	OR (CI 95 %)	*P*-value
Study Phase						
Phase 1	reference	-	reference	-	reference	-
Phase 2	2.92 (1.35–6.31)	<0.01	3.01 (1.41–6.38)	<0.01	3.14 (1.72–5.73)	<0.001
Age	0.93 (0.92–0.96)	<0.001	0.94 (0.92–0.96)	<0.001	0.95 (0.94–0.97)	<0.001
ISS	0.93 (0.91–0.96)	<0.001	0.94 (0.92–0.96)	<0.001	0.96 (0.94–0.98)	<0.001
GCS	1.24 (1.12–1.38)	<0.001	1.29 (1.16–1.43)	<0.001	1.2 (1.11–1.29)	<0.001
Pupils						
None reactive	Reference	-	Reference	-	Reference	-
One reactive	9.89 (2.12–45.9)	<0.01	7.57 (1.75–32.6)	<0.01	4.03 (1.09–14.85)	<0.01
Both reactive	6.66 (2.58–17.1)	<0.001	6.09 (2.42–15.32)	<0.001	4.95 (1.97–12.45)	<0.001
Midline shift						
>5 mm	Reference	-	Reference	-	reference	-
<5 mm	0.55 (0.17–1.74)	0.309	0.58 (0.18–1.81)	0.348	2.07 (0.71–6)	0.18
No shift	1.92 (0.79–4.66)	0.149	1.56 (0.66–3.71)	0.314	5 (2.26–11)	<0.001
Ringers lactate administered	1.56 (0.62–3.97)	0.348	2.01 (0.83–4.89)	0.124	1.44(0.69–3.01)	0.319
Ringers solution administered	1.25 (0.49–3.12)	0.635	1.51 (0.63–3.6)	0.358	0.93 (0.45–1.91)	0.843
Colloids administered	0.58 (0.13–2.41)	0.447	0.6 (0.15–2.42)	0.472	0.9 (0.26–3.14)	0.869
EMS arrival to admission time <60 min	1.49 (0.61–3.71)	0.384	2.29 (0.95–5.49)	0.063	0.87 (0.42–1.8)	0.715
Admission to first CT time <60 min	1.39 (0.59–3.3)	0.441	1.14 (0.49–2.65)	0.761	1.54 (0.79–3.04)	0.203

Legend: *ISS* Injury Severity Score, *GCS* Glasgow Coma Scale, *EMS* Emergency Medical Service, *OR* Odds Ratio, *CI* Confidence Interval, *ICU* Intensive Care Unit

^a^The models used also center as a predictor, thus the presented results took the ‘center effect’ into account (actual OR and *p*-values are not listed in the table due to large space required)

## Discussion

To the best of our knowledge this is the first project that tried to identify the most important treatment options, summarized these in a short document, and began implementing them. The results demonstrated that outcomes of patients with TBI can be improved if the recommended treatment options are used more frequently. Predicted mortality and predicted rate of unfavorable outcome were identical in both phases. There were no other differences in treatment options (e.g., increased use of tranexamic acid etc.). Thus, we believe the significantly better outcomes observed in Phase 2 were probably due to the achieved improvements in early TBI care.

The impact of time factors after TBI seems to be well known; yet there is conflicting evidence, whether duration (“golden hour”) or quality of emergency care has more influence on the outcome of TBI patients [20]. Some studies suggest that direct transport to an appropriate center is associated with improved outcomes [21]. The importance of in-hospital time factors has been shown in other studies, too [22, 23]. Our project successfully significantly reduced rates of secondary transfers and significantly increased proportion of those patients who got to the hospital within 60 min from EMS arrival. Regarding the time intervals hospital admission – ‘first CT in less than 60 min after admission’ and ‘from hospital admission to operation room in less than 120 min’ the project was less successful; the improvements were statistically not significant.

The use of capnography in patients who need prehospital intubation has been shown to reduce the rate of hyperventilation [24]. It has also been shown that end-tidal CO_2_ correlates well with ventilation in patients with isolated TBI [25]. However, the same study showed that end-tidal pCO_2_ shows poor correlation to ventilation in patients with major trauma and shock. This is due to the fact that end-tidal pCO_2_ may be related to pulmonary perfusion rather than to ventilation in these states. Therefore, the target range of end-tidal pCO_2_ has to be chosen according to the status of the trauma patient. Our recommendations included a target range of 30–35 mmHg (corresponding to an arterial pCO_2_ of 35–40 mmHg in patients with normal perfusion). Our data show that the use of capnography reduced the rate of hyperventilation. This part of the project was implemented successfully.

Fluid resuscitation is an important option in patients with TBI. Resuscitation with isotonic crystalloids is considered standard care. It has been shown that resuscitation using albumin is associated with worse outcomes in TBI patients [26], and that hypertonic saline is not superior to normal saline [27] in hypotensive patients with TBI. Our recommendations were to avoid hypotonic crystalloids (e.g., Ringer’s lactate), use isotonic crystalloids, and to add colloids and/or hypertonic saline in hypotensive patients. These recommendations were followed in the majority of patients.

The recommendation to use TEM as early as possible to optimize coagulation in all patients >60 years and/or on anticoagulants is a new aspect. This recommendation was based on the finding that during Phase 1, patients who had had TEM in the Trauma Room had higher hospital survival (51 % vs. 38 %) and a higher rate of favorable outcome (40 % vs. 28 %). A number of recent studies suggest that the use of TEM to guide coagulation management may be beneficial after multiple trauma [28, 29] as well as after TBI [30]. The significant increase in early use of TEM during Phase 2 may have contributed to the better outcomes of the Phase 2 patients.

The recommendation not to use steroids was made because during the first Phase 15 patients received corticosteroids; the mortality of these patients was significantly higher (15 % vs. 0 %) – thus, the use of corticosteroids more than doubled the odds for mortality. The recommendations not to use corticosteroids was one of the few “standards” in the 1996 BTF guidelines [12], and is still a “Level 1” recommendation in the 2007 version. The CRASH trial [31] has shown that use of corticosteroids is associated with increased mortality. It is unclear why corticosteroids were used so frequently during Phase 1; only 1/15 patients had a concomitant spinal cord injury.

Regarding epidemiology, >70 % of the patients were male; this is the typical male: female ratio for moderate and severe TBI [32]. The mean age was rather high; almost one third of the patients were > 65 years old. This mirrors a clear trend during the last decades: a high rate of geriatric TBI has been observed in other European studies, too [9]. Thus, traffic accidents were a leading cause of injury, but same-level falls (i.e., the typical mechanism of TBI in geriatric patients) were the cause of accident in almost one third of all cases.

## Limitations

It is important to address the study limitations. The hospital selection may have introduced bias as the treatment centers participated in our study based on their own decision upon the invitation.

Despite the thorough monitoring we were not able to prevent missing data in the prehospital setting and outcome follow-up. Loss to follow-up is prominent mainly in Phase 1 with 44 % of unknown outcome. The main reason was the absence of contact information and that was better taken care of in Phase 2.

## Conclusions

In conclusion, in a study done in 14 Austrian centers we have identified prehospital and early hospital treatment options that were potentially associated with better outcomes. Based on these results, recommendations were drafted and implemented with the support of participating hospitals and EMS. These recommendations dealt with time factors, monitoring and treatment options. We were able to demonstrate that these recommendations led to reduction of time delays and improved quality of care, both pre- and in-hospital in Phase 2 of our study. Patients treated in participating treatment centers after the implementation of recommended changes had three-fold chance of survival and favorable long-term outcome.

Thus, improvement in early TBI care was followed by a significant improvement in patient outcomes.

## Abbreviations

AIS: Abbreviated Injury Scale
AUVA: Austrian Worker’s Compensation Board
BTF: Brain Trauma Foundation
CI: Confidence interval
CO2: Carbon dioxide
CT: Computed Tomography
EMS: Emergency medical services
GCS: Glasgow Coma Scale
GOSE: Extended Glasgow Outcome Scale
ICU: Intensive care unit
IQR: Interquartile range
ICP: Intracranial pressure
ISS: Injury Severity Score
INRO: International Neurotrauma Research Organization
O/E: Observed versus expected
OR: Odds Ratio
p CO2: Partial pressure of carbon dioxide
SD: Standard Deviation
TEM: Thrombelastometry
TR: Trauma Room
TBI: Traumatic Brain Injury
